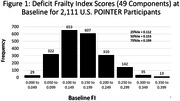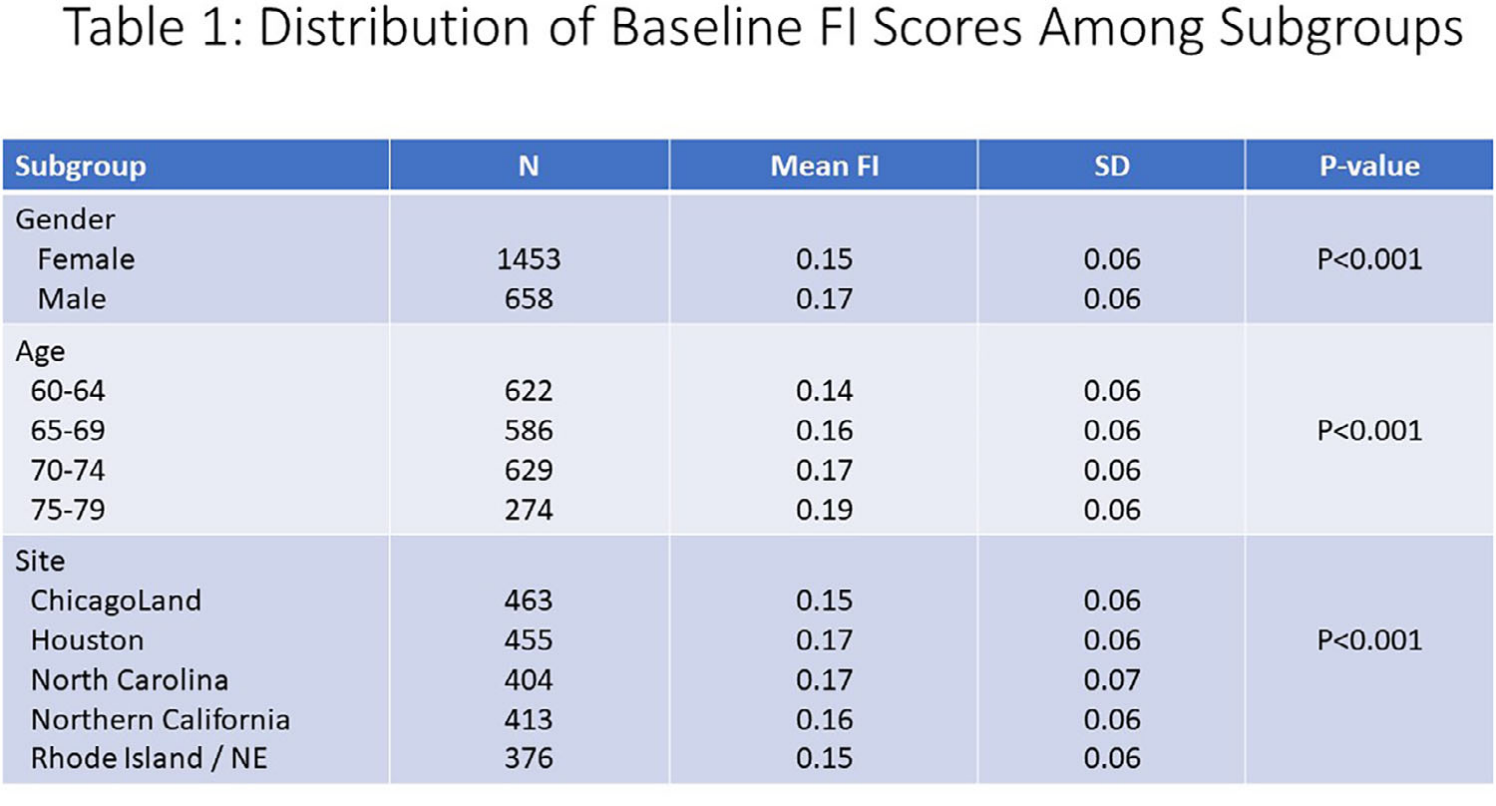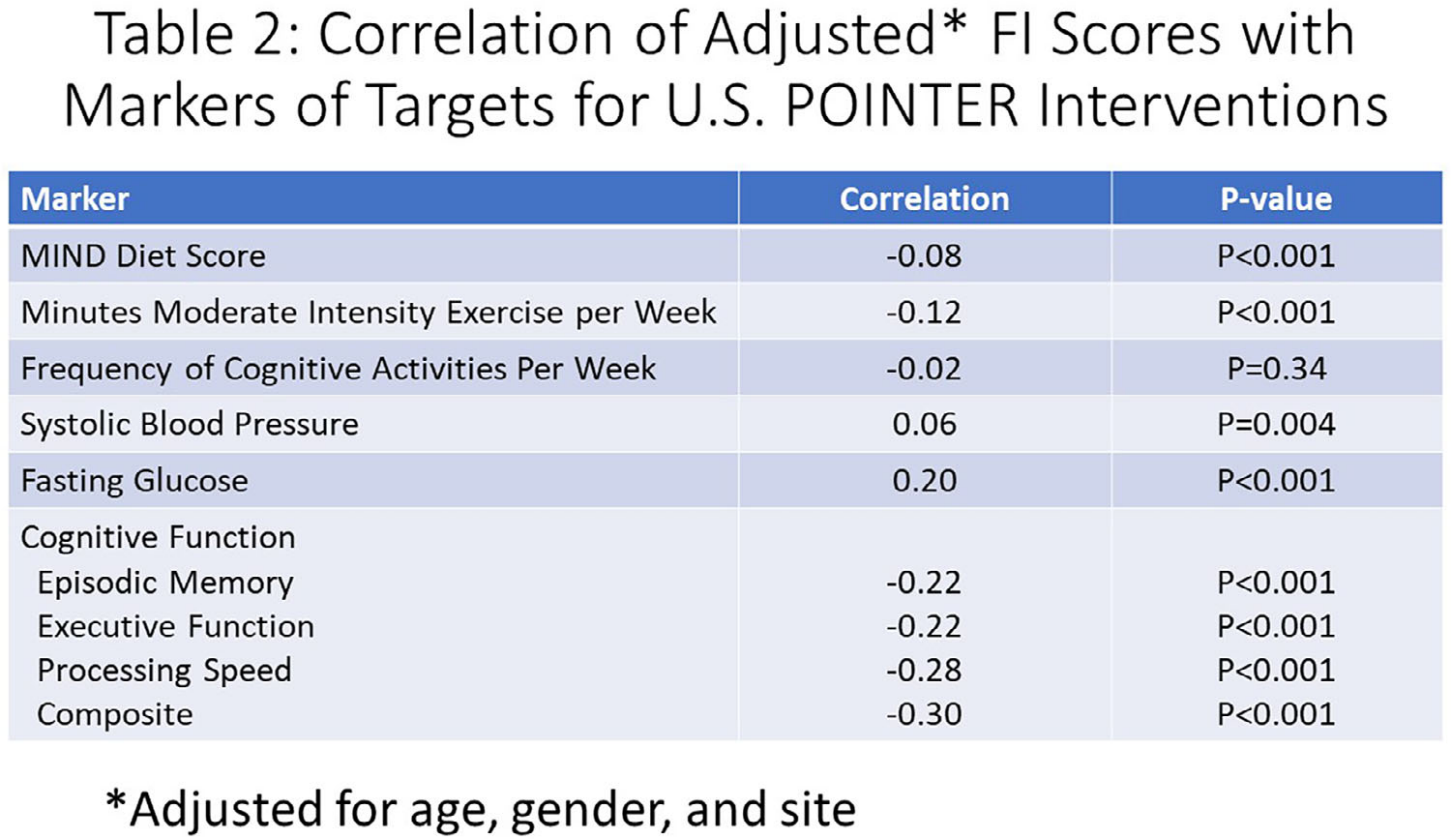# Associations Between Deficit Accumulation Frailty and Baseline Markers of Lifestyle and Health in the U.S. POINTER Trial

**DOI:** 10.1002/alz.088205

**Published:** 2025-01-09

**Authors:** Mark A. Espeland, Yitbarek N Demesie, Maryjo Cleveland, Sarah Tomaszewski Farias, Laura D Baker, Lucia Crivelli, Katelyn R Garcia, Samuel N. Lockhart, KayLoni Olson, Heather M Snyder, Christy C Tangney, Rena R. Wing, Rachel A. Whitmer, Michelle York, Kathryn E. Callahan, U.S. POINTER Study Group

**Affiliations:** ^1^ Wake Forest University School of Medicine, Winston Salem, NC USA; ^2^ Wake Forest University School of Public Health, Winston‐Salem, NC USA; ^3^ Wake Forest University School of Medicine, Winston‐Salem, NC USA; ^4^ University of California, Davis School of Medicine, Sacramento, CA USA; ^5^ Wake Forest University, Winston‐Salem, NC USA; ^6^ Fleni, Buenos Aires, Buenos Aires Argentina; ^7^ kayloni_olson@brown.edu, Providence, RI USA; ^8^ Alzheimer’s Association, Chicago, IL USA; ^9^ Rush University, Chicago, IL USA; ^10^ Miriam Hospital, Providence, RI USA; ^11^ Baylor, Houston, TX USA

## Abstract

**Background:**

Behavioral interventions designed to promote healthy lifestyles have the potential to slow biological aging and increase health span. Multidomain interventions that simultaneously target multiple lifestyle behaviors may particularly be promising by increasing the number of interrelated processes that might be benefited. Deficit accumulation frailty indices (FIs) are increasingly used as measures of aging and health status in clinical trials and cohort studies.

**Method:**

A 49‐component FI was developed from baseline measures of age‐related 1) physical function and abilities, 2) cognitive function, 3) health related quality of life, 4) mood and affect, 5) clinical biomarkers, 6) physical measures, 5) sleep quality, 8) chronic diseases, 9) sensorineural abilities, 10) polypharmacy, and 11) behaviors. The FI was calculated for the 2,111 participants of the U.S. Study to Protect Brain Health through Lifestyle Intervention to Reduce Risk (U.S. POINTER), a two‐armed randomized controlled clinical trial comparing two multidomain lifestyle interventions on cognitive function. Associations between the FI and markers of intervention targets were assessed using correlation coefficients after covariate adjustment for age, gender, and clinical site.

**Result:**

The 25%ile, 50%ile, and 75%ile of the FI distribution (Figure 1) were 0.112, 0.153, and 0.199 and are consistent with a cohort at risk for accelerated aging. Mean FI scores were greater by 13% among males compared with females and by 36% among those aged 75‐79 years compared with those aged 60‐64 years (Table 1; both p<0.001). Greater FI scores were associated with worse MIND diet scores, fewer minutes of moderate exercise per week, higher systolic blood pressures, higher fasting glucose concentrations, and worse domain‐specific and composite cognitive function (all p≤0.01) but were unrelated to the frequency of cognitive activities per week (Table 2).

**Conclusion:**

The U.S. POINTER FI has expected associations with targets of its multidomain lifestyle interventions. It is designed to serve as a basis for assessing whether these interventions differentially slow aging and, if so, how this may contribute to better cognitive functioning.